# Mechanism study on the sulfidation of ZnO with sulfur and iron oxide at high temperature

**DOI:** 10.1038/srep42536

**Published:** 2017-02-10

**Authors:** Junwei Han, Wei Liu, Tianfu Zhang, Kai Xue, Wenhua Li, Fen Jiao, Wenqing Qin

**Affiliations:** 1 School of Minerals Processing and Bioengineering, Central South University, 410083, Changsha, Hunan, China

## Abstract

The mechanism of ZnO sulfidation with sulfur and iron oxide at high temperatures was studied. The thermodynamic analysis, sulfidation behavior of zinc, phase transformations, morphology changes, and surface properties were investigated by HSC 5.0 combined with FactSage 7.0, ICP, XRD, optical microscopy coupled with SEM-EDS, and XPS. The results indicate that increasing temperature and adding iron oxide can not only improve the sulfidation of ZnO but also promote the formation and growth of ZnS crystals. Fe_2_O_3_ captured the sulfur in the initial sulfidation process as iron sulfides, which then acted as the sulfurizing agent in the late period, thus reducing sulfur escape at high temperatures. The addition of carbon can not only enhance the sulfidation but increase sulfur utilization rate and eliminate the generation of SO_2_. The surfaces of marmatite and synthetic zinc sulfides contain high oxygen due to oxidation and oxygen adsorption. Hydroxyl easily absorbs on the surface of iron-bearing zinc sulfide (Zn_1−*x*_Fe_*x*_S). The oxidation of synthetic Zn_1−*x*_Fe_*x*_S is easier than marmatite in air.

Most of nonferrous metals, such as Cu, Pb and Zn, are primarily recovered from sulfide ores with beneficiation followed by metallurgical process^1^. With the ceaseless exploitation of metal resources, high-grade ores are exhausted day by day, and correspondingly, millions of tons of smelting wastes containing plenty of heavy metals are generated every year in the world^2^. In the past decades, most users preferred stockpiling or landfilling to recycling the wastes because of lack of economic and legislative driving forces. Since the heavy metals in wastes are rarely in sulfides but are in oxides and oxidized compounds, which are more soluble in water than their sulfide counterparts, the concerns are not only the waste of metal resources but environmental threats^3^,^4^. For catering to the sustainable development of nonferrous industry, a number of hydrometallurgical^5^,^6^,^7^,^8^, pyrometallurgical^9^,^10^ and their combined processes^11^,^12^,^13^,^14^ have been performed to exploit low-grade oxide ores and recycle valuable metals from smelting wastes. Despite many achievements made, these technologies have still not been widely applied for mass production due to the presence of some technical and economical drawbacks.

Sulfidation has recently received much attention as a possible generic technology for the recovery of valuable metals from low-grade oxide ores or wastes. In this process, metal oxides and oxidized compounds are converted into sulfides, which have a good floatability and are relatively insoluble in aqueous solutions^15^. As a result, the aim to recover metals by flotation and reduce the pollution of heavy metals can be achieved theoretically. At present, many sulfidation methods have been proposed to convert various oxidized materials, including sulfidation with Na_2_S^16^,^17^, mechanochemical sulfidation^18^,^19^, hydrothermal sulfidation^20^,^21^,^22^,^23^ and sulfidation roasting^24^,^25^. Sulfidation roasting is more beneficial for the formation and growth of sulfide crystals and thus shows better results for the recovery of nonferrous metals by flotation. Li *et al*.^26^ investigated the recovery of lead and zinc from low-grade Pb-Zn oxide ore by sulfidation roasting and flotation process. The results indicated that the sulfidation degree of lead and zinc reached 98% and 95%, respectively. Meanwhile, 79.5% Pb and 88.2% Zn were recovered by conventional flotation, and the concentrate contained 10.2% Pb and 38.9% Zn. Wang *et al*.^27^ studied the sulfidation roasting and flotation of cervantite. A flotation concentrate grading 21.04% Sb with a recovery of 77.15% was achieved. Zheng *et al*.^28^,^29^,^30^ employed sulfidation roasting and flotation process to recycle valuable metals from lead smelter slag and zinc leaching residues. The experimental results of zinc leaching residue showed that a flotation concentrate with 39.13% Zn, 6.93% Pb and 973.54 g/t Ag was obtained, and the recovery rates of Zn, Pb and Ag were 48.38%, 68.23% and 77.41%, respectively. By contrast, it is more difficult to recover valuable metals from the smelter slag, because it contains complex amorphous phases, resulting in the metal sulfides generated with low crystallinity and fine grains.

Base on the fact that increasing temperature can not only accelerate chemical reactions but also promote the formation and growth of crystals, some researchers attempted to use a high-temperature roasting process to convert the metal oxides into sulfides with sufficient particle size and good crystalline structure. Harris *et al*.^31^,^32^ investigated the sulfidation of nickeliferous lateritic ore with sulfur. They found that at low temperatures the Fe-Ni-S phase formed was submicron in nature and heating to temperature between 1050 and 1100 °C not only allowed for the growth of the particles, but also facilitated the further reaction of iron sulfides with nickel oxides to iron oxides and nickel sulfides, which was conducive to the enrichment of nickel by flotation. Han *et al*.^25^,^33^ carried out the selective sulfidation of lead smelter slag at high temperatures. The results indicated that, although the selective transformation of lead smelter slag could be achieved and the zinc sulfides with coarse grains and good crystallinity were generated after sulfidation roasting at above 1000 °C, a qualified zinc sulfide concentrate has never been obtained. The reasons are complicated and from many aspects, which needs to be resolved. However, the majority of studies on sulfidation roasting have been restricted to the investigation in process optimization. Additionally, numerous studies focused on the sulfidation of ZnO nanoparticles for synthesizing ZnO/ZnS core–shell nanostructures by hydrothermal process at low or room temperatures. The mechanism of these studies is obviously different from that of our study^34^,^35^,^36^,^37^,^38^,^39^,^40^. Therefore, it is necessary to establish a system of theoretical knowledge for further developing the sulfidation roasting technology of metal oxides.

In the current study, the sulfidation behavior of ZnO roasted with sulfur at high temperatures was systematically studied for the first time. Since sulfur is volatile and easy to escape at high temperatures, the sulfidation would be first carried out at 400 °C to avoid sulfur loss and then performed at a higher temperature for further sulfidation and the particle growth and crystal modification of the metal sulfides generated. Meanwhile, iron oxide (typically Fe_2_O_3_) was used as an additive to capture sulfur in the initial stage, and the iron sulfides obtained would act as the sulfurizing agent in the late period of the roasting at high temperatures. In this paper, the thermodynamic analysis, sulfidation behavior of zinc, phase transformations, morphology changes, and surface properties were investigated by HSC 5.0 combined with FactSage 7.0, ICP, XRD, optical microscopy coupled with SEM-EDS, and XPS, respectively. The purpose was to clarify the mechanisms of ZnO sulfidation with sulfur and sulfur capture with iron oxide at high temperatures and thus to provide a theoretical foundation for guiding the development of sulfidation roasting process.

## Experimental Section

### Materials

Zinc oxide (ZnO), sulfur (S), and ferric oxide (Fe_2_O_3_) are of analytical grade and were purchased from Sinopharm Chemical Reagent Co., Ltd. in China. The marmatite sample used for XPS analysis was obtained from Dachang dressing plant, Guangxi (China). It contains 49.73% Zn, 27.90% S, and 12.43% Fe. A carbon powder containing 53% C was used as the reducing agent. All samples used were ground and sieved to smaller than 74 μm for the experiments.

### Methods

The sulfidation roasting was performed in an elevator furnace, whose schematic was given in the previous paper^33^. For each test, 10 g ZnO powder was thoroughly mixed with sulfur, Fe_2_O_3_ and carbon powders in a scheduled mass ratio. The mixture was loaded in an alundum crucible with a volume of 100 mL and sealed with a cover followed by iron wire bundling. The alundum crucible was then put into the furnace. Prior to the roasting, a nitrogen gas (N_2_) with a flow speed of 2 L/min was introduced into the furnace for excluding air. Thereafter, the mixture was heated at a rate of 40 °C/min to 400 °C and held at this temperature for 2 h, and then further heated to a required temperature for 1 h. When the roasting finished, the roasted mixture was taken out after they were cooled to room temperature under N_2_ atmosphere, then weighed, ground, and analyzed by a selective leaching and ICP for the sulfidation degree of zinc, whose detailed analysis process and calculation method were described by Han *et al*.^33^.

In this study, the zinc content of samples was determined with inductively coupled plasma (ICP, IRIS Intrepid II XSP). The crystal phase compositions were analyzed by X-ray powder diffraction (XRD, Germany Bruker-axs D8 Advance). The morphological characteristics were analyzed by optical microscopy (Leica DMRXP) and scanning electron microscopy (SEM, Quanta FEG250) combined with energy dispersive spectroscopy (EDS, Genesis XM2). Both powder and lump samples were used for the morphological analysis, thus each of the powder samples was made into a lump with a polished surface in advance, through bonding, cutting, grinding, and polishing processes^41^. Additionally, X-ray photoelectron spectroscopy (XPS) study was carried out with a Thermo Scientific ESCALAB 250Xi using an Al Kα X-ray source. Binding energy calibration was based on C 1 s at 284.8 eV. The background of the spectrum was obtained using the Shirley method. A nonlinear least-square curve-fitting program (Avantage software 5.52) was used to deconvolve the XPS data.

## Results and Discussion

### Thermodynamic analysis

**Figure 1:**
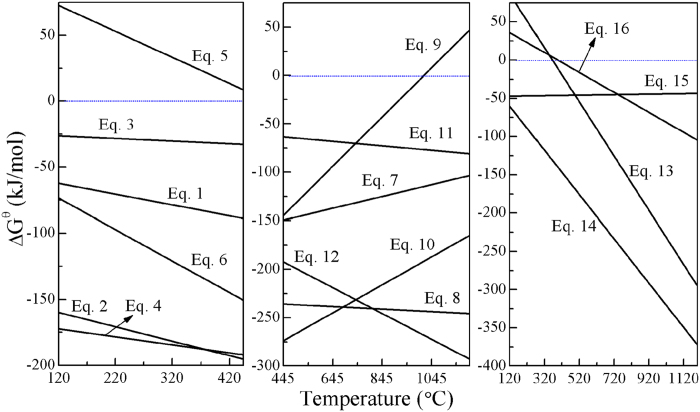
Standard Gibbs free changes of the possible reactions as a function of temperature in the range of 120–1200 °C.

As is well known, the melting point and boiling point of sulfur are approximately 120 °C and 445 °C, respectively. Therefore, the possible sulfidation reactions of ZnO and Fe_2_O_3_ with sulfur at the temperature range of 120–445 °C are as follows:


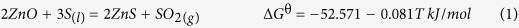



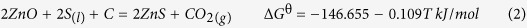



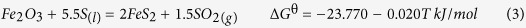



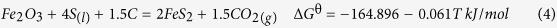



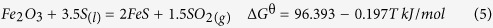



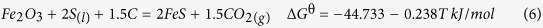


where the *∆G*^θ^*−T* equations of these reactions were obtained by data-fitting using Origin 8.0, and the primary data of standard Gibbs free energy changes (∆*G*^θ^) for these reactions at the range of 120 to 445 °C were calculated by HSC Chemistry 5.0 (Fig. 1). It is found that ZnO and Fe_2_O_3_ can react with liquid sulfur to ZnS and FeS_2_ or FeS at the range of 120 to 445 °C, except for reaction (5). Obviously, the addition of carbon not only promotes these sulfidation reactions but also can increase sulfur utilization rate and eliminate the generation of SO_2_, indicating that carbon plays a positive role in the sulfidation of metal oxides.

In the sulfidation roasting, the objective of preheating at 400 °C is to convert elemental sulfur into zinc and iron sulfides as much as possible for reducing sulfur loss by evaporation. However, it is needed to introduce a high-temperature roasting process for the further sulfidation of ZnO and the growth of the ZnS crystals generated. The ∆*G*^θ^ for the possible sulfidation reactions of ZnO and Fe_2_O_3_ with sulfur gas (S_2_) at the range of 445 to 1200 °C were calculated, as well. Meanwhile, the reactions of ZnO with iron sulfides (mainly FeS_2_ and FeS) at the range of 120 to 1200 °C were also investigated. The equations of these reactions are given as follows:


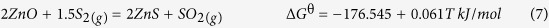



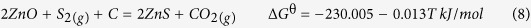



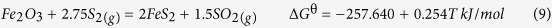



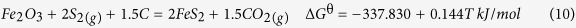



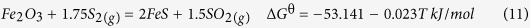



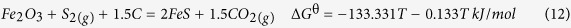



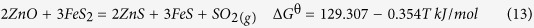



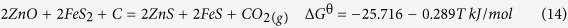



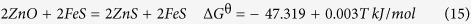



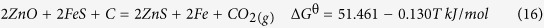


The functions of *∆G*^θ^*−T* for reactions (7) to (16) were also drawn in Fig. 1. The results reveal that the sulfidation of ZnO and Fe_2_O_3_ with S_2_ are thermodynamically feasible, except for reaction (9) at above 1014 °C. The *∆G*^θ^ of reactions (7) to (12) are respectively lower than that of reactions (1) to (6) in most of the temperature range, suggesting that high-temperature roasting not only promotes the growth of ZnS fine particles but also favors sulfidation reactions both thermodynamically and kinetically. It is also found from Fig. 1 that ZnO can react with FeS_2_ and FeS above 400 °C, demonstrating that the captured sulfur can act as sulfidizing agent to further transform ZnO into ZnS, when sulfur is insufficient in the late period of the roasting. Meanwhile, the addition of carbon also favors these reactions. In addition, the sulfidation of ZnO with sulfur or iron sulfides in the presence of carbon can be promoted by increasing temperature. As a result, the selective sulfidation of zinc and iron oxides with sulfur can be achieved by roasting with carbon at high temperatures. Hence, the sulfidation roasting were carried out at the temperature range of 800 to 1200 °C.

**Figure 2:**
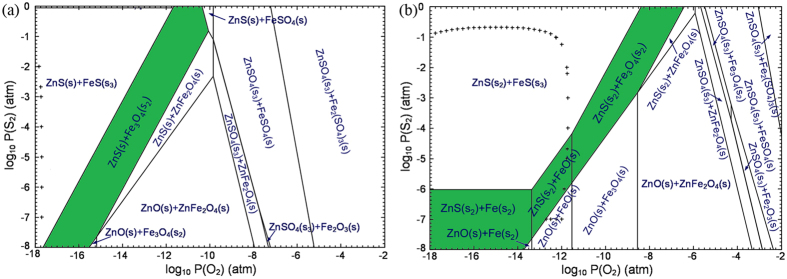
Predominance-area diagrams of Fe-Zn-S-C-O system at (**a**) 800 °C and (**b**) 1100 °C. [0.333 < Zn/(Fe + Zn) < 1, log_10_ P(CO) = −1 (atm), ‘+’ = 1.0 atm P(total) isobar)].

On the other hand, the selective sulfidation was also investigated with the predominance-area diagrams of Fe-Zn-S-C-O system (Fig. 2), which were calculated using the Predom module of FactSage 7.0^42^. The results indicate that the selective sulfidation to obtain zinc sulfide and iron nonsulfides, which favors the separation of zinc and iron by conventional flotation, can be achieved by adjusting temperature and the partial pressures of S_2_ and O_2_, based on the presence of the desired stability areas (marked with green) in the predominance-area diagrams of Fe-Zn-S-C-O system. Compared with 800 °C, the predominance-area diagram at 1100 °C shows a larger ZnS stability area and a smaller ZnFe_2_O_4_ area (ZnFe_2_O_4_ is an undesired mineral phase but is usually generated in a smelting process), indicating that increasing temperature is beneficial to the sulfidation of ZnO. Besides, the target region of selective sulfidation expands to a higher range of O_2_ partial pressure as the temperature increases from 800 to 1100 °C. Therefore, high temperature is helpful for reducing the requirement of reductive atmosphere. Based on the thermodynamic analysis, the selective sulfidation of ZnO roasted with sulfur and iron oxide can be achieved under a reductive condition at high temperatures.

**Figure 3:**
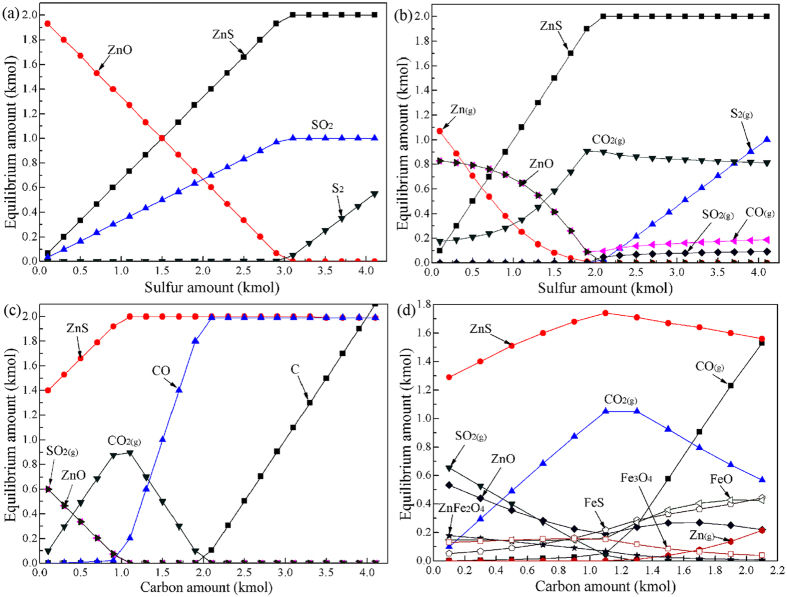
Equilibrium phase diagrams of ZnO sulfidation at 1100 °C with (**a**) different sulfur amount, (**b**) different sulfur amount and 1 kmol carbon, (**c**) different carbon amount and 2 kmol sulfur, and (**d**) different carbon amount, 2 kmol sulfur and 0.5 kmol Fe_2_O_3_.

To further investigate the thermodynamics of ZnO sulfidation, the equilibrium compositions of the reaction products at 1100 °C were calculated using the Equilibrium Compositions module of HSC 5.0 and determined by the Gibbs free energy minimization method for isothermal, isobaric and fixed mass conditions. The detailed calculation method referred to the papers by Pickles *et al*.^43^,^44^,^45^. The effect of sulfur dosage on the equilibrium amounts of possible species was studied. The ZnO amount was fixed at 2 kmol. The results for without carbon and with 1 kmol carbon are presented in Fig. 3a and b, respectively. It is found that the addition of carbon not only promotes ZnO sulfidation and reduces the sulfur amount required, but also can eliminate the generation of SO_2_. This is consistent with the conclusion from Fig. 1. With the increase in sulfur dosage, ZnS amount gradually increases until its maximum at 3.1 kmol S for without carbon or at 2.1 kmol S for with 1 kmol carbon. Further increasing sulfur dosage, S_2_ gas begins to appear and increases significantly. In addition, SO_2_ is regenerated when sulfur amount is more than 2 kmol. Hence, the optimal sulfur dosage is considered as 2 kmol. Figure 3c and d show the effects of carbon dosage on the sulfidation of ZnO without and with 0.5 kmol Fe_2_O_3_, respectively. It concluded that 1.1 kmol is the optimum carbon dosage depending on the considerations from ZnS amount, SO_2_ amount and carbon utilization rate. For the addition of Fe_2_O_3_, with the increase of carbon dosage from 0.1 kmol to 1.1 kmol, FeS amount gradually decreases as ZnS amount increases. However, above 1.1 kmol carbon the situation is reversed. On the whole, the selective sulfidation of ZnO reacted with sulfur and iron oxide can be achieved, although some FeS and ZnFe_2_O_4_ are still remained under the optimized conditions, which may be a problem caused by the addition of Fe_2_O_3_.

### Sulfidation behavior of ZnO

**Figure 4:**
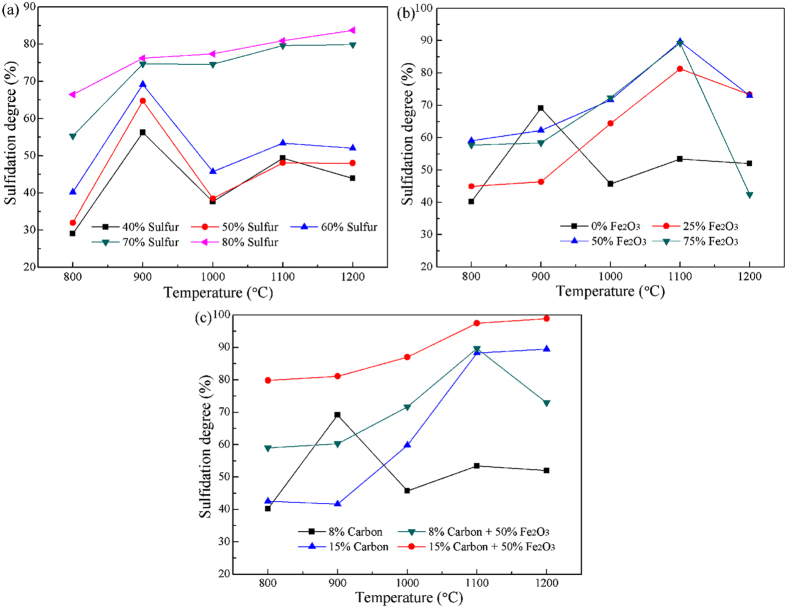
Effects of roasting parameters on the sulfidation behavior of ZnO at different temperatures: (**a**) sulfur dosage (carbon dosage was fixed at 8 wt.%), (**b**) Fe_2_O_3_ dosage (sulfur and carbon dosage were fixed at 60 wt.% and 8 wt.%, respectively), and (**c**) carbon dosage (sulfur dosage were fixed at 60 wt.%).

Although the thermodynamic analysis of ZnO sulfidation was studied and some significant conclusions have been obtained, it is essential to investigate the sulfidation behavior of ZnO by a series of experiments. The effects of temperature, sulfur dosage, Fe_2_O_3_ dosage, and carbon dosage on the sulfidation of ZnO were investigated based on the thermodynamic analysis (Fig. 4). It can be found from Fig. 4a that the sulfidation of ZnO can be promoted by increasing temperature, but the higher roasting temperature the more sulfur required. For sulfur dosage within 60%, the ZnS generated was reoxidized due to the fast consumption of sulfur at high temperatures. To limit the loss of sulfur, Fe_2_O_3_ was introduced to the sulfidation process for capturing sulfur in advance, as mentioned previously. As shown in Fig. 4b, without the addition of Fe_2_O_3_, the sulfidation degree of zinc increases with the increase from 800 to 900 °C, above which it begins to decrease. However, with the addition of Fe_2_O_3_, the sulfidation degree increases gradually until the temperature above 1100 °C. This indicates that Fe_2_O_3_ could capture sulfur in the initial period of the roasting and then the generated iron sulfides acted as the sulfurizing agent in the late period of the sulfidation. With the increase in Fe_2_O_3_ dosage, the sulfidation degree of ZnO increases significantly, but when Fe_2_O_3_ dosage is more than 50%, the value has no significant variation, except for the temperature above 1100 °C, indicating that 50% Fe_2_O_3_ is sufficient for the sulfidation. Above 1100 °C, the sulfidation degree of zinc decreases as the temperature further increases, and the decrease extent significantly increases with the increase in Fe_2_O_3_ dosage, because high temperature and the addition of Fe_2_O_3_ can accelerate the consumption of carbon, resulting in that it is insufficient above 1100 °C. The carbon dosage was therefore increased for attempting to improve the utilization rate of sulfur. The results shown in Fig. 4c reveal that increasing carbon dosage promoted the sulfidation of ZnO and thus improved the utilization rate of sulfur, no matter whether or not adding Fe_2_O_3_. The sulfidation degree of ZnO reached 97.5% after the roasting with 60% sulfur, 15% carbon and 50% Fe_2_O_3_ at 1100 °C. These conclusions drawn from the roasting experiments are in accordance with those obtained by thermodynamic analysis.

### Phase transformations

**Figure 5:**
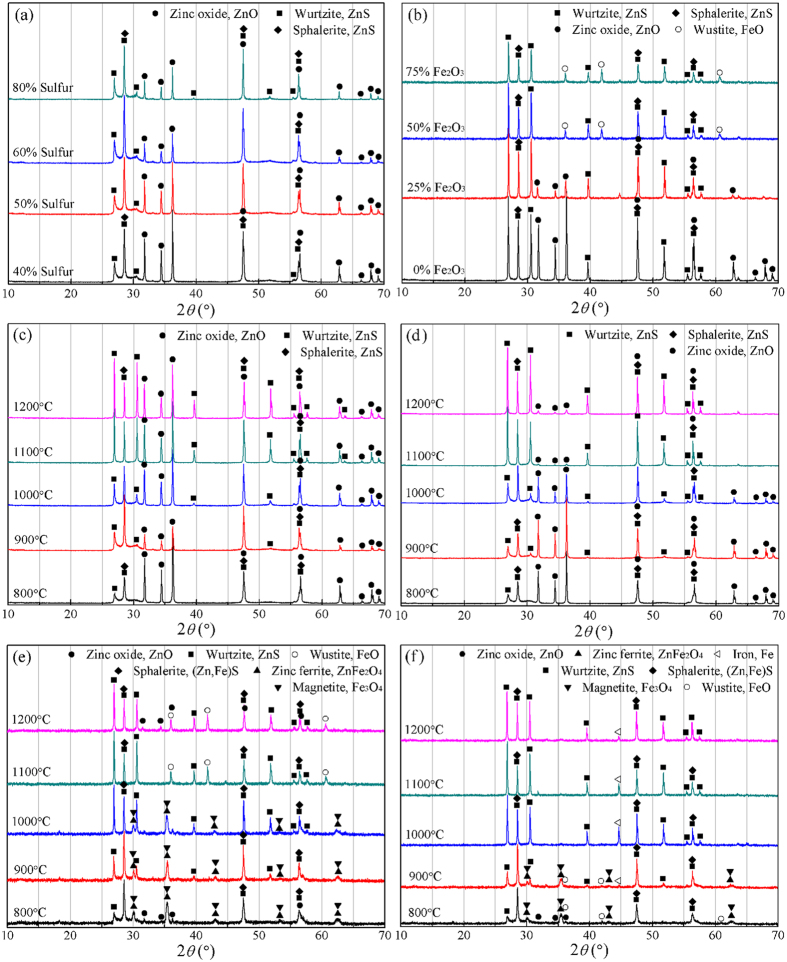
XRD patterns of the samples roasted with (**a**) different sulfur dosage at 900 °C, (**b**) different Fe_2_O_3_ dosage at 1100 °C, (**c**) 60% sulfur and 8% carbon, (**d**) 60% sulfur and 15% carbon, (**e**) 50% Fe_2_O_3_ and 8% carbon, and (**f**) 50% Fe_2_O_3_ and 15% carbon.

To investigate the phase transformation behaviors during the sulfidation process, all of the samples roasted previously for the investigation on the sulfidation behavior of ZnO were analyzed by XRD and some of the results are given in Fig. 5. It is seen from Fig. 5a that the diffraction peaks of ZnS (sphalerite plus wurtzite) gradually increase, while the peaks of ZnO decrease, as the sulfur dosage increases. Obviously, increasing sulfur dosage promoted the sulfidation of ZnO, but ZnO peaks always presented in the XRD patterns, which indicated that the transformation of ZnO to ZnS was incomplete. As shown in Fig. 5b, the addition of Fe_2_O_3_ was helpful for the sulfidation of ZnO and the optimized dosage was considered as 50%, under which the peaks of ZnO disappeared, while the wustite was generated. Figure 5c and d show the XRD patterns of the samples roasted with 60% sulfur at different temperatures in the presence of 8% and 15% carbon, respectively. It is found that the changes for ZnS peaks intensity are consistent with the corresponding sulfidation degree variations in Fig. 4(c). For 8% carbon, ZnO amount was significantly reduced while sphalerite was markedly increased as the temperature increased from 800 to 900 °C, above which some ZnS generated were reoxidized due to insufficient sulfur and carbon and thus the amount of sphalerite was decreased. However, wurtzite was increased gradually with the increase from 800 to 1200 °C, which reveals that increasing temperature can promote the transformation of sphalerite to wurtzite, especially above 1000 °C. For 15% carbon, with the increase in temperature, the intensity of ZnO decreased and that of ZnS increased. When the temperature reached 1100 °C, the peaks of ZnO were hardly observed. Hence, increasing carbon dosage is conducive to the sulfidation of ZnO. Figure 5e and f present the XRD patterns of the samples roasted with 60% sulfur and 50% Fe_2_O_3_ at different temperatures in the presence of 8% and 15% carbon, respectively. The changes for ZnS peaks intensity well confirmed the corresponding data of the sulfidation degree in Fig. 4(c). It is found that the increase both in temperature and carbon dosage not only promoted the sulfidation but also affected the iron mineral phase. For 8% carbon, the iron was mainly in the form of Fe_3_O_4_ and ZnFe_2_O_4_ within 1000 °C, above which they were converted into FeO. For 15% carbon, the iron primarily exists as Fe_2_O_3_, ZnFe_2_O_4_ and FeO within 900 °C, above which it was mainly in the form of metallic iron, which is strongly magnetic and thus can be separated by magnetic separation.

**Figure 6:**
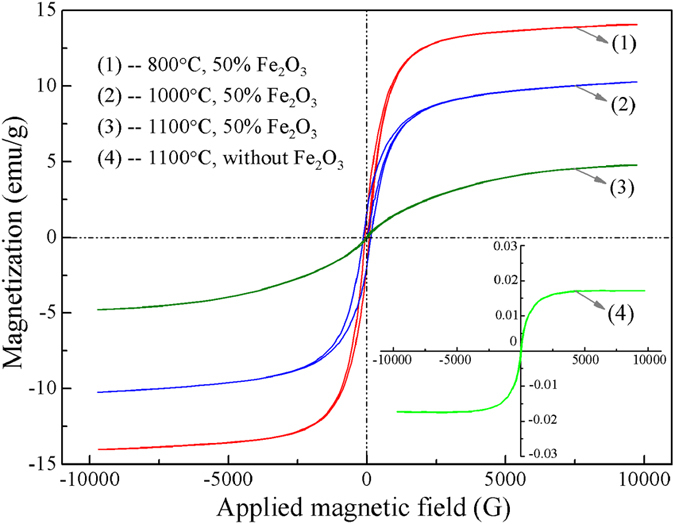
Magnetic hysteresis loops of the samples roasted with 60% sulfur and 8% carbon at different temperatures.

It is difficult to distinguish ZnFe_2_O_4_ and Fe_3_O_4_ by XRD because all the peaks of them are almost overlapped, thus the samples roasted with Fe_2_O_3_ at different temperatures were detected by Vibration Sample Magnetometer (BHV-50HTI) (Fig. 6). The results indicated that Fe_3_O_4_ was generated after the roasting and its amount decreased with the increase of temperature from 800 to 1100 °C, based on the changes of saturation magnetization. Besides, note that some of iron contained in ZnS crystal lattices as solid solution. In conclusion, carbon dosage of 15% is better than 8% for the sulfidation of ZnO. Since the carbon powder used contains 53% C, the carbon of 15% actually contains about 8% C, which is the theoretical value of the carbon required, based on the thermodynamic analysis.

### Microscopic morphology changes

**Figure 7:**
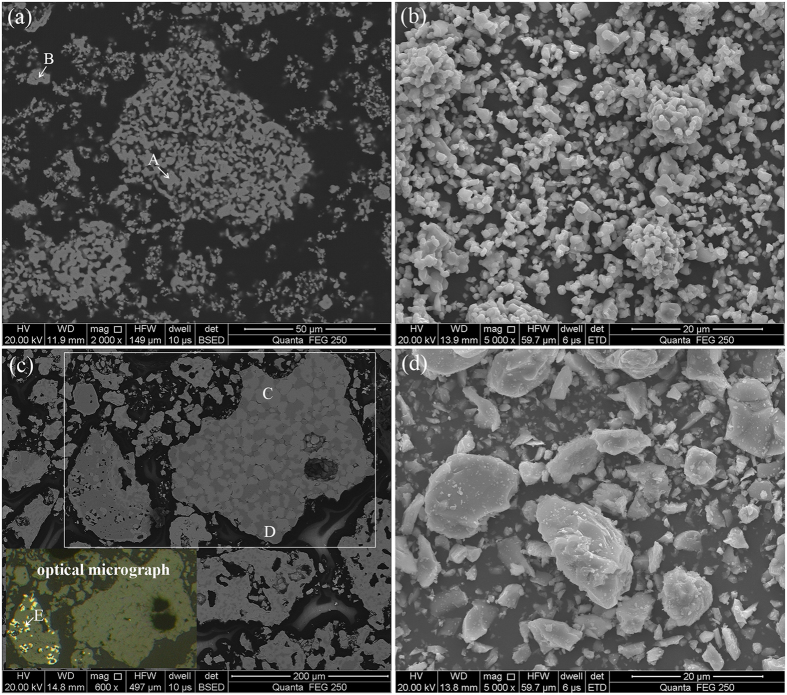
SEM images and optical micrograph of the samples roasted with 60% sulfur and 8% carbon at 1100 °C: (**a**) without additive (for lump), (**b**) without additive (for powder), (**c**) with 50% Fe_2_O_3_ (for lump), and (**d**) with 50% Fe_2_O_3_ (for powder).

The samples roasted without and with Fe_2_O_3_ were investigated by optical microscopy and SEM-EDS (Fig. 7). Both lump and powder samples were subjected to SEM-EDS analysis. It is seen from Fig. 7a and b that the sample roasted without Fe_2_O_3_ is fine, porous, and no clear boundary between different mineral phases. EDS analysis indicated that it was mainly composed of ZnS and ZnO (Table 1), which confirmed the XRD results. By contrast, the sample roasted with 50% Fe_2_O_3_ has a larger particle size and more compact structure. Most of the mineral phases in the sample can be distinguished from the others (Fig. 7c), and the ZnS particles generated have clear edges and corners (Fig. 7d). Therefore, the addition of Fe_2_O_3_ not only improved the sulfidation of ZnO but also promoted the formation and growth of ZnS crystals, because the generation of the intermediates with low melting points (iron sulfides) improved the liquidity of the reactants^32^. On the other hand, the majority of ZnO was converted into iron-bearing zinc sulfides and thus ZnO was hardly found by EDS, but some zinc reacted with iron oxide to zinc-bearing magnetite (Zn_*x*_Fe_3−*x*_O_4_), which is a spinel, magnetic and insoluble in mild solution, and is usually produced during the reduction process of zinc ferrite^46^,^47^,^48^, was generated. The spinel formation is bad for the separation of zinc and iron, but it can be decreased by increasing the reducibility of the reaction system. This could be proved by the fact that the diffraction peaks of ZnFe_2_O_4_ and Fe_3_O_4_ (In this paper, Zn_*x*_Fe_3−*x*_O_4_ is considered as the mixtures of ZnFe_2_O_4_ and Fe_3_O_4_ with different ratio) were significantly decreased and even disappeared with the increase in temperature and carbon dosage, as presented in Fig. 5. In addition, it is found that some of Fe_2_O_3_ was converted to metallic iron. Hence, high temperature and sufficient carbon are conducive to the sulfidation of ZnO.

### Surface property analysis

**Figure 8:**
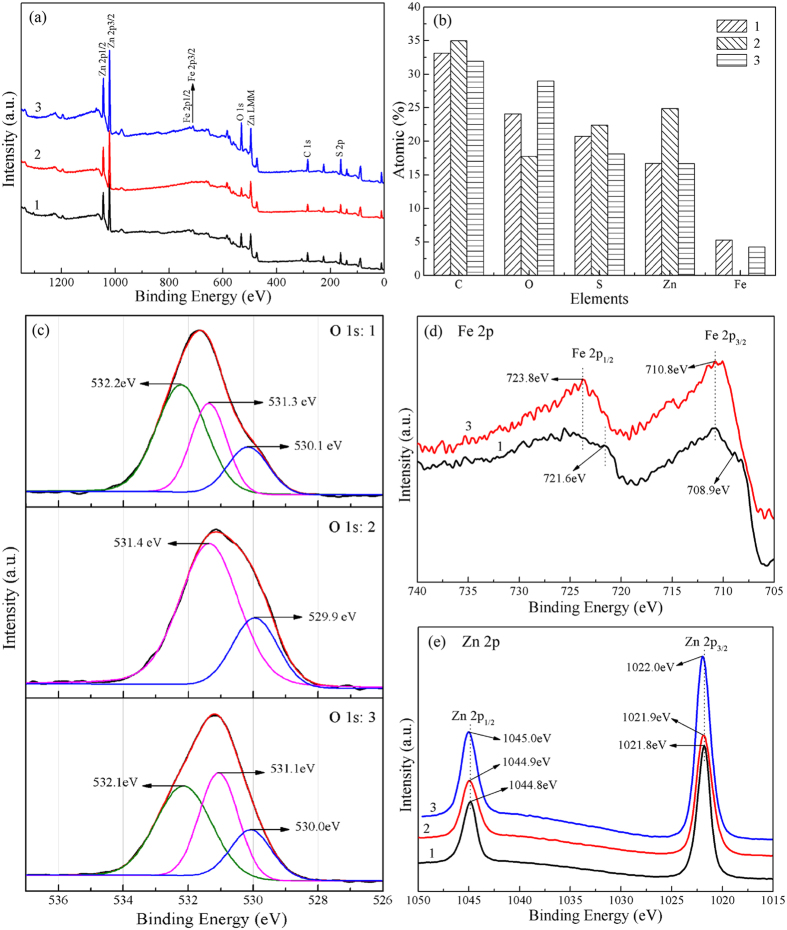
Surface properties of natural marmatite (1), the ZnO roasted with 70% sulfur (2), and the ZnO roasted with 60% sulfur and 50% Fe_2_O_3_ (3): (**a**) XPS survey spectra, (**b**) chemical compositions on the surface of these samples, and high-resolution scans for (**c**) O 1 s, (**d**) Fe 2p, and (**e**) Zn 2p electrons.

The surface elemental composition and chemical status of natural marmatite, the ZnO roasted with 70% sulfur (synthetic ZnS), and the ZnO roasted with 60% sulfur and 50% Fe_2_O_3_ (synthetic Zn_1−*x*_Fe_*x*_S) were investigated by XPS. A wide survey scan of XPS spectra was taken in the range of 0–1350 eV (Fig. 8a). The peaks of Zn, Fe, S, and O are observed from the surfaces of these samples, except for Fe that is not on the surface of synthetic ZnS. Note that C was introduced onto the surface of the samples in XPS analysis and thus has not been discussed. As shown in Fig. 8b, the atomic percentages of O on the surfaces of these samples are very high, which is attributed to various oxidation and oxygen adsorption on their surfaces, based on the differences in the position and shape of O 1 s peaks among the three samples (Fig. 8c). The O 1 s signal of synthetic ZnS could be deconvolved into two Gaussian fitted peaks with the binding energies of 531.4 eV and 529.9 eV, which were ascribed to the surface-adsorbed oxygen species such as O^−^ and the surface lattice oxygen (O^2−^) in a zinc/iron-oxide framework^49^,^50^,^51^,^52^,^53^. For synthetic Zn_1−*x*_Fe_*x*_S, the O 1 s signal was resolved into three peaks at 532.1 eV, 531.1 eV, and 530.0 eV. The peaks locate at 532.1 was assigned to absorbed hydroxyl oxygen^54^,^55^,^56^, and the others could respectively correspond to the oxygen species existed on the surface of synthetic ZnS. The original O 1 s signal of marmatite was deconvolved into three peaks at 532.2 eV, 531.3 eV, and 530.1 eV, each peak of which respectively corresponded to the oxygen species on the surface of synthetic Zn_1−*x*_Fe_*x*_S. The presence of lattice oxygen species suggest that the sulfidation of ZnO with sulfur relates to the migration of oxygen from the inside of ZnO to its surface^24^,^57^,^58^, and that the surface of marmatite can be oxidized in air. It is also found that hydroxyl easily absorbs on the surface of iron-bearing zinc sulfide, in comparison with the zinc sulfide without Fe. This may be a reason that marmatite is more difficult than sphalerite to be recovered by flotation. Figure 8d and e reveals that the high-resolution spectra of Fe 2p and Zn 2p for marmatite and synthetic Zn_1−*x*_Fe_*x*_S, respectively. According to literatures^59^,^60^, the Fe 2p_3/2_ peaks at 710.8 eV and 708.9 eV signify the presence of Fe^3+^ and Fe^2+^, respectively. Since the iron contained in marmatite is Fe^2+^, the results demonstrate that natural marmatite and synthetic Zn_1−*x*_Fe_*x*_S can be oxidized from their surfaces in air, which may be another reason that marmatite is more difficult than sphalerite to be floated with xanthate^61^,^62^,^63^, and the oxidation of synthetic Zn_1−*x*_Fe_*x*_S is easier than marmatite. This may be a reason that natural ZnS is easier than synthetic ZnS to be floated^64^,^65^. The Zn 2p peaks of the roasted samples only have slight shift compared with that of marmatite (1021.8 eV)^66^, indicating that most of ZnO has been converted into ZnS after the sulfidation roasting.

**Figure 9:**
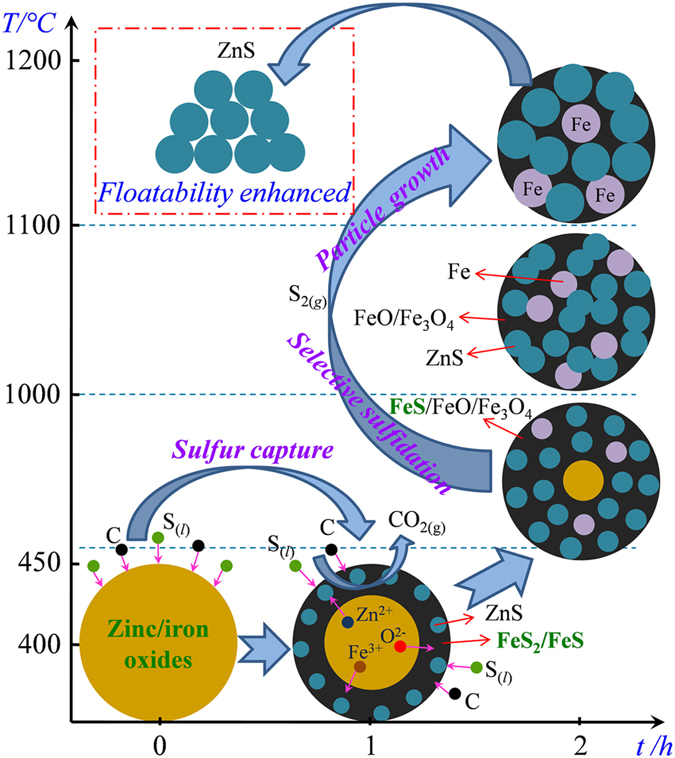
Schematic diagram for the mechanism of ZnO sulfidation with sulfur and iron oxide.

According to the above analyses, the mechanisms of ZnO sulfidation with sulfur and sulfur capture with iron oxide during the developed roasting process are summarized in Fig. 9.

## Conclusions

The thermodynamic and experimental investigations indicate that increasing temperature can not only enhance the reaction of ZnO with sulfur but also favor ZnS particle growth and the conversion of sphalerite to wurtzite, especially above 1000 °C. However, sulfur is volatile and easy to escape at high temperatures. Fe_2_O_3_ captured the sulfur in the initial sulfidation process as iron sulfides, which then acted as the sulfurizing agent in the late period, thus the utilization rate of sulfur was increased. The ZnO roasted without Fe_2_O_3_ is fine and no clear boundaries between different mineral phases, but the sample roasted with 50% Fe_2_O_3_ has a larger particle size, mineral phases are easily distinguished from others, and the ZnS particles generated have clear edges and corners. Hence, the addition of Fe_2_O_3_ not only improved the sulfidation of ZnO but promoted the formation and growth of ZnS crystals, because of the generation of the intermediates with low melting points. Carbon plays an important role in ZnO sulfidation. The addition of carbon not only promoted the sulfidation but also could increase sulfur utilization rate and eliminate the generation of SO_2_. XPS analysis reveals that the surfaces of marmatite and synthetic zinc sulfides contain high oxygen due to oxidation and oxygen adsorption. Hydroxyl easily absorbs on the surface of iron-bearing zinc sulfide (Zn_1−*x*_Fe_*x*_S). The oxidation of synthetic Zn_1−*x*_Fe_*x*_S is easier than marmatite in air. It is also found that the sulfidation of ZnO with sulfur relates to the migration of oxygen from the inside of ZnO to its surface. This study should contribute to a fundamental knowledge that can be used to guide the sulfidation roasting of zinc-containing materials, which is missing in the literatures at the present time.

## Additional Information

**How to cite this article**: Han, J. *et al*. Mechanism study on the sulfidation of ZnO with sulfur and iron oxide at high temperature. *Sci. Rep.* **7**, 42536; doi: 10.1038/srep42536 (2017).

**Publisher's note:** Springer Nature remains neutral with regard to jurisdictional claims in published maps and institutional affiliations.

## Figures and Tables

**Table 1:** Chemical composition of the selected points in SEM images by EDS.

Points	Element content (wt.%)				
	Zn	Fe	S	O	C
A	66.64	—	28.99	0.58	3.79
B	84.72	—	3.01	9.02	3.25
C	63.15	4.75	31.42	0.68	—
D	19.02	65.34	0.18	15.46	—
E	—	100	—	—	—
